# Overview of Witloof Chicory (*Cichorium intybus* L.) Discolorations and Their Underlying Physiological and Biochemical Causes

**DOI:** 10.3389/fpls.2022.843004

**Published:** 2022-02-23

**Authors:** Isabel De Jaegere, Yannah Cornelis, Tim De Clercq, Alain Goossens, Bram Van de Poel

**Affiliations:** ^1^Laboratory of Molecular Plant Hormone Physiology, Division of Crop Biotechnics, Department of Biosystems, University of Leuven, Leuven, Belgium; ^2^Praktijkpunt Landbouw Vlaams-Brabant, Herent, Belgium; ^3^Department of Plant Biotechnology and Bioinformatics, Ghent University, Ghent, Belgium; ^4^VIB Center for Plant Systems Biology, Ghent, Belgium

**Keywords:** chicory, witloof, quality, discolorations, polyphenol oxidase

## Abstract

Many fruits and vegetables suffer from unwanted discolorations that reduce product quality, leading to substantial losses along the supply chain. Witloof chicory (*Cichorium intybus* L. var. *foliosum*), a specialty crop characterized by its unique bitter taste and crunchiness, is particularly sensitive to various types of red and brown discolorations. The etiolated vegetable suffers from three predominant color disorders, i.e., core browning, internal leaf reddening, and leaf edge browning. Additionally, several less frequently observed color disorders such as hollow pith, external red, and *point noir* can also negatively affect crop quality. In this article, we bring together fragmented literature and present a comprehensive overview of the different discoloration types in chicory, and discuss their potential underlying physiological causes, including laticifer rupture, calcium deficiency, and a disturbed water distribution. We also describe the role of environmental cues that influence discoloration incidence, including cultivation and postharvest storage conditions such as forcing and storage temperature, root ripeness and the duration of the forcing process. Finally, we zoom in on the underlying biochemical pathways that govern color disorders in witloof chicory, with a strong emphasis on polyphenol oxidase.

## Introduction

Witloof chicory (*Cichorium intybus* L. var. *foliosum*, syn. Belgian endive, chicon) is a leafy vegetable with firm white-yellow leaves and a characteristic bitter taste. In Europe, chicory has a long history of cultivation for its taproot and leaves, but the particular method that produces the etiolated buds known as witloof chicory, was only introduced in the 19th century in the region of Brussels in Belgium (van Kruistum and Zwanepol, 1997; Lucchin et al., 2008). Today, production of witloof chicory is mainly localized in Western Europe, with the Netherlands, Belgium, France, Italy, and Germany as main producers. However, the global market is rapidly expanding, as chicons are exported to distant markets such as the United States, Canada, Japan, Qatar, and Israel, adding to a world trade value of 102 million United States dollar in 2019 (OEC, 2021). New markets, such as the United States, are ramping up their local witloof chicory production (Corey et al., 1990), highlighting the growing international interest in this specialty bitter vegetable.

Chicory is a member of the *Asteraceae* family, more specifically the *Cichorieae* tribe, which is typified by the presence of milky latex and homogamous ligulate capitula and includes well-known relatives such as lettuce and dandelion (Kilian et al., 2009). Within the species, *C. intybus* shows a broad genetic diversity, harboring a wide range of different ecotypes and numerous cultivated varieties (Lucchin et al., 2008; Ghanaatiyan and Sadeghi, 2016, 2017). In accordance with their different uses, chicory cultivars can be divided into three subtypes, i.e., root chicory, leaf chicory, and witloof chicory, which show a clear genetic diversion (Raulier et al., 2016). Due to its biennial life cycle, witloof chicory is produced in two stages. During the first, vegetative stage, the crop is sown during late spring in open fields to produce a rosette of green leaves with a fleshy taproot, which is harvested in the late fall and stored at low temperature to initiate generative growth. The duration of the root storage period thereby depends on the cultivar and its subsequent forcing window, during which chicon yield and quality are optimal. During the second stage, the vernalized taproot is stimulated to produce an etiolated apical bud in a dark, humid environment; a process known as forcing. Traditionally, growers covered the taproots with soil to induce chicon growth, but today, most witloof chicory is grown hydroponically in a multilayer system in dark growth chambers (van Kruistum and Zwanepol, 1997). The combination of hydroponic forcing systems, together with the introduction of hybrid cultivars and a well-balanced long-term root storage program, enables chicon production all year round (de Proft et al., 2003).

Witloof chicory is typically subjected to rigorous quality standards regarding shape, firmness, and visual appearance. Chicons should be symmetrical with tightly-packed turgid leaves tapering to a pointed tip. The leaves are cream-colored with smooth, yellow edges and free of any pigmentation by chlorophyll. The occurrence of physiological disorders such as brown or red discolorations on either the leaves or pith can largely reduce the economic value and may diminish shelf life, nutritional value, and consumer appreciation (Yoruk and Marshall, 2003). Although discolorations already occurred in traditionally grown soil chicory, a lot of color defects arose upon the introduction of high-yielding cultivars bred specifically for hydroponic systems (Den Outer, 1989; Reerink, 1994). Color defects are still pertinent nowadays, considering that 10–50% of chicons can suffer discolorations after postharvest storage (personal communication Praktijkpunt Landbouw Vlaams-Brabant). Past studies have described the issue of discolorations in witloof chicory but profound fundamental knowledge about the exact causes of these physiological disorders is largely lacking. In this review, we bring together the current knowledge to categorize and illustrate the different types of discolorations in witloof chicory, and we provide insight into the physiological and biochemical factors that govern these disorders.

## Overview of Discoloration Types

Unwanted discolorations in chicons of witloof chicory can be grouped into three major types i.e., leaf reddening, leaf edge browning, and pith disorders. In addition, a number of less frequent discoloration types may occur as a result of specific environmental or postharvest storage conditions, including *point noir* and chilling injury. Here, we illustrate and describe the different color disorders and briefly present the underlying physiological causes. More details on the factors influencing color disorders and the biochemical processes causing discolorations, will be presented in the next section.

### Leaf Reddening

A common quality disorder in chicory involves the development of red discolorations on some of the leaves. A distinction is made between internal red and external red, based on the localization of the symptoms and their occurrence throughout the forcing season.

#### Internal Red

**Figure 1:**
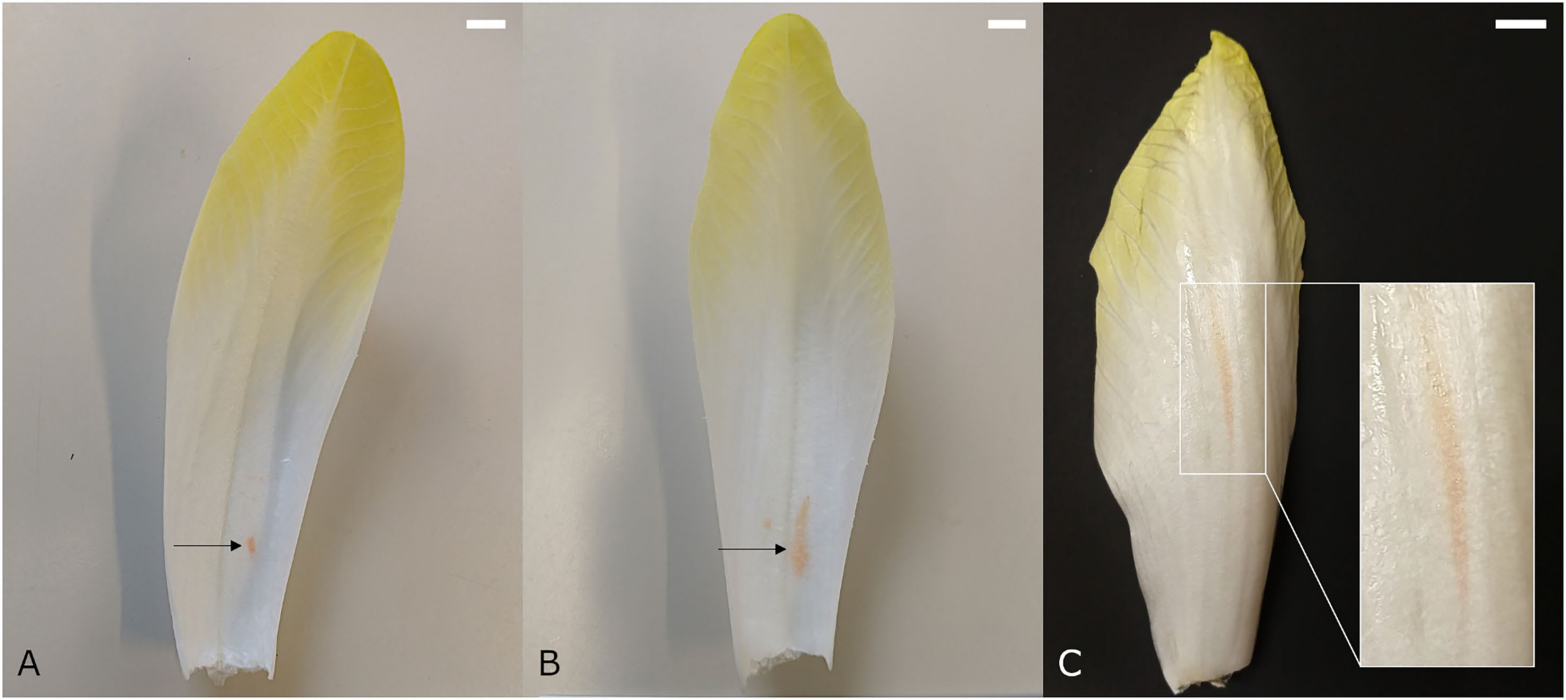
Medial chicon leaf showing internal red discoloration with **(A)** initial and **(B)** more advanced symptoms. **(C)** Chicory leaf with external red discoloration symptoms. Scale bars represent 1 cm.

Red discolorations that occur adaxially on the basal part of the leaves are referred to as internal red. This color disorder occurs predominantly during late forcing (spring) and develops mostly postharvest. It is initiated by the appearance of crisp red spots on the lower half of the midrib of medial leaves. In a later stage, the red color may spread further into the leaf lamina, resulting in a red haze throughout the basal part of the leaf (Reerink, 1994; Figures 1A,B). Veen (1999) and Coppenolle et al. (2001) pointed out that internal red is associated with gaps that are formed between the adaxial epidermis and the subepidermal parenchyma cell layers. After harvest, the continued growth of the flower stalk or pith may attribute to the progression of this red lesion by extracting water and perhaps also nutrients from the leaves, as well as exerting mechanical stress on the tightly packed medial leaves.

#### External Red

Whereas internal red occurs predominantly during late forcing, external reddening manifests mainly during early forcing (autumn). External red occurs on the outer leaves of the chicon, showing elongated red spots on the abaxial side that run parallel to the vascular bundles (Reerink, 1994; van Kruistum and Zwanepol, 1997; Figure 1C). The spots are diffuse and occur mostly on the basal part of the leaf, but can extend toward the tip. External red presumably develops when forcing temperatures are relatively high at the start of the forcing season, which leads to rapid elongation of the leaves during the last days of forcing. The high rate of water uptake associated with a fast cellular elongation can cause laticifer cells to rupture. This results in leakage of latex into surrounding tissue and the subsequent development of discolorations. Asymmetric turgor pressure differences between laticifers and the surrounding parenchyma cells, as well as weakened cell wall structures due to calcium deficiency, have been proposed to play a role in the development of external red (Reerink, 1994).

### Pith Disorders

Several discoloration types can develop in the pith, which is the central core of the chicon that develops into a flowering stem when allowed to grow in the light. The most studied pith disorder is core browning, but other physiological defects such as apple pith, pink axis, and hollow pith also occur on a regular basis.

#### Core Browning

**Figure 2:**
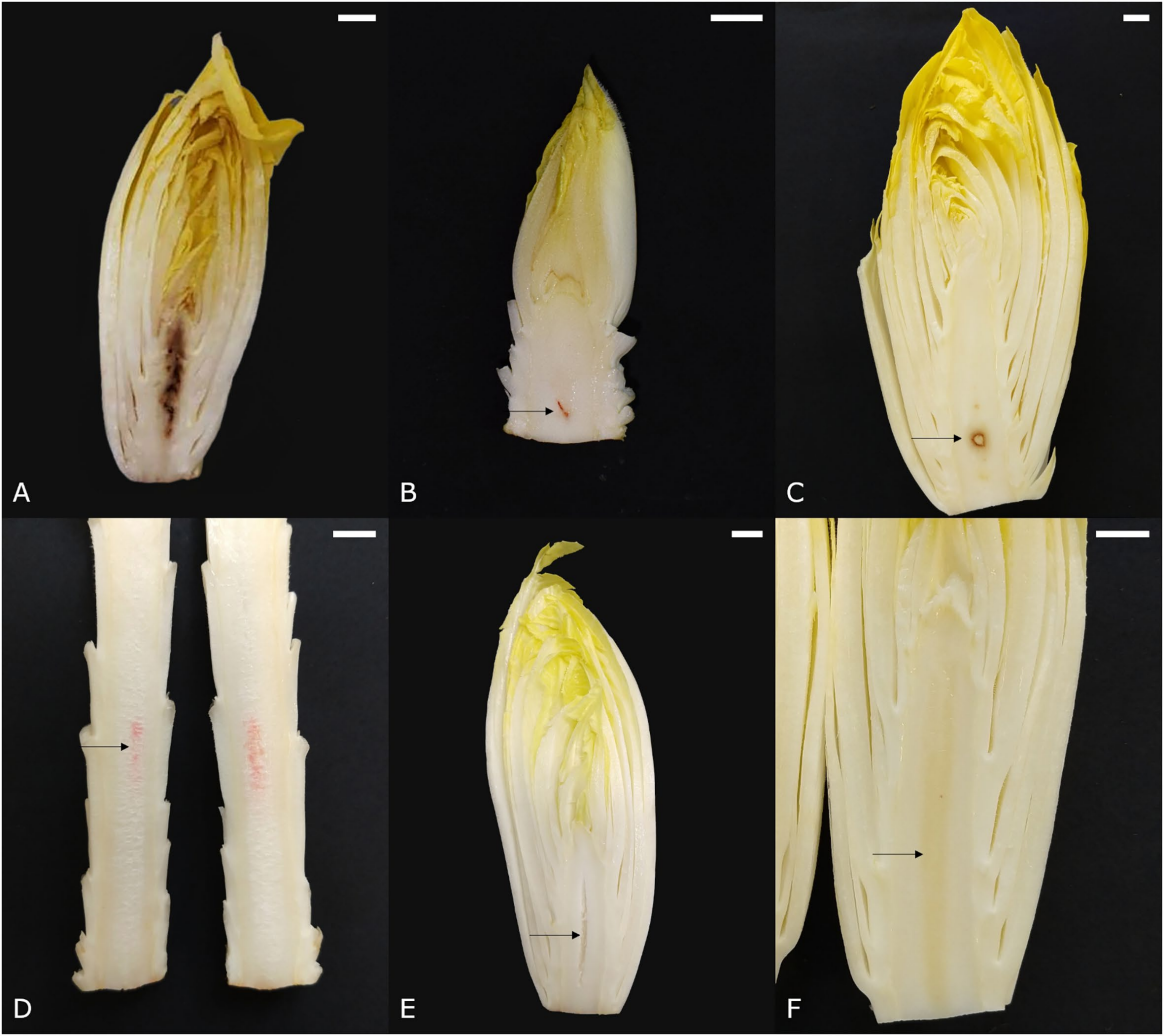
Symptoms of pith discolorations in witloof chicory. **(A)** Spotted core browning, **(B)** layered core browning, **(C)** apple pith, **(D)** porous pith with pink discoloration, **(E)** hollow pith, and **(F)** glassy pith. Scale bars represent 1 cm.

Core browning (syn. brown pith, internal browning) appears as brown or dark red discolorations in the core parenchyma. The first symptoms arise during forcing and can gradually aggravate as the pith continues to grow during postharvest storage. A brown pith can occur in two distinct forms. The first type involves spot-like core browning, in which the core tissue turns translucent and subsequently yellow-brown to dark brown due to an increasing loss of cellular integrity (de Barsy and Bronchart, 1991; Figure 2A). The second type involves the discoloration of several layers of pith tissue and is referred to as layered core browning (Figure 2B). According to Den Outer (1989), layered core browning is associated with a disturbed cellular organization in the central core due to irregular cell division. Later, cells undergo plasmolysis, followed by the discoloration of the cell content, and finally cellular collapse. Around the discolored core tissue, series of radial cells are produced that separate the affected region from the healthy surrounding pith tissue. It is thought that core browning is associated with an inadequate supply of calcium during chicon development. A lack of calcium can lead to disintegration of the middle lamellae and the detachment of cell walls, which causes a gradual loss of structural integrity and eventually cellular collapse (Den Outer, 1989; de Barsy and Bronchart, 1991).

#### Apple Pith

The name of this disorder refers to its resemblance to the cross-section of an apple seed. Apple pith designates an area of opaque white tissue that is sharply delineated by a dark red-brown border, usually situated around the center of the core near the base (Figure 2C). In severe cases, the affected central tissue may collapse, leaving only the colored outer edge and thus a hollow pith (van Kruistum and Zwanepol, 1997). Symptomatic similarities between apple pith and the sharply delineated discolorations observed in layered core browning, make it plausible that apple pith is a more severe manifestation of core browning.

#### Pink Axis

Sometimes, a pink discoloration can be seen along the longitudinal axis of the pith (Figure 2D). It usually develops during postharvest storage, especially when the pith is very elongated. Symptoms vary in intensity from a nearly indistinguishable haze to a bright pink stripe.

#### Textural Pith Disorders

Several other physiological pith disorders, including porous, hollow—and glassy pith, are not necessarily color disorders but rather textural defects of the core parenchyma tissue. A porous pith (Figure 2D) shows core tissue that is sponge-like and has an opaque white appearance, as opposed to the glossy white appearance of healthy pith tissue. Typically, a porous pith develops postharvest and is often seen in combination with a pink axis (Figure 2D). Hollow pith designates a cavity that usually occurs along the central axis and can take on a number of different forms, depending on the physiological process that caused the cavity (Figure 2E). This disorder occurs quite frequently and can reduce product quality, especially when visible from the base. A glassy pith (Figure 2F), is characterized by a yellow and vitreous appearance of the core, similar to the initial stage of spotted core browning, and is often overlooked.

### Leaf Edge Browning

**Figure 3:**
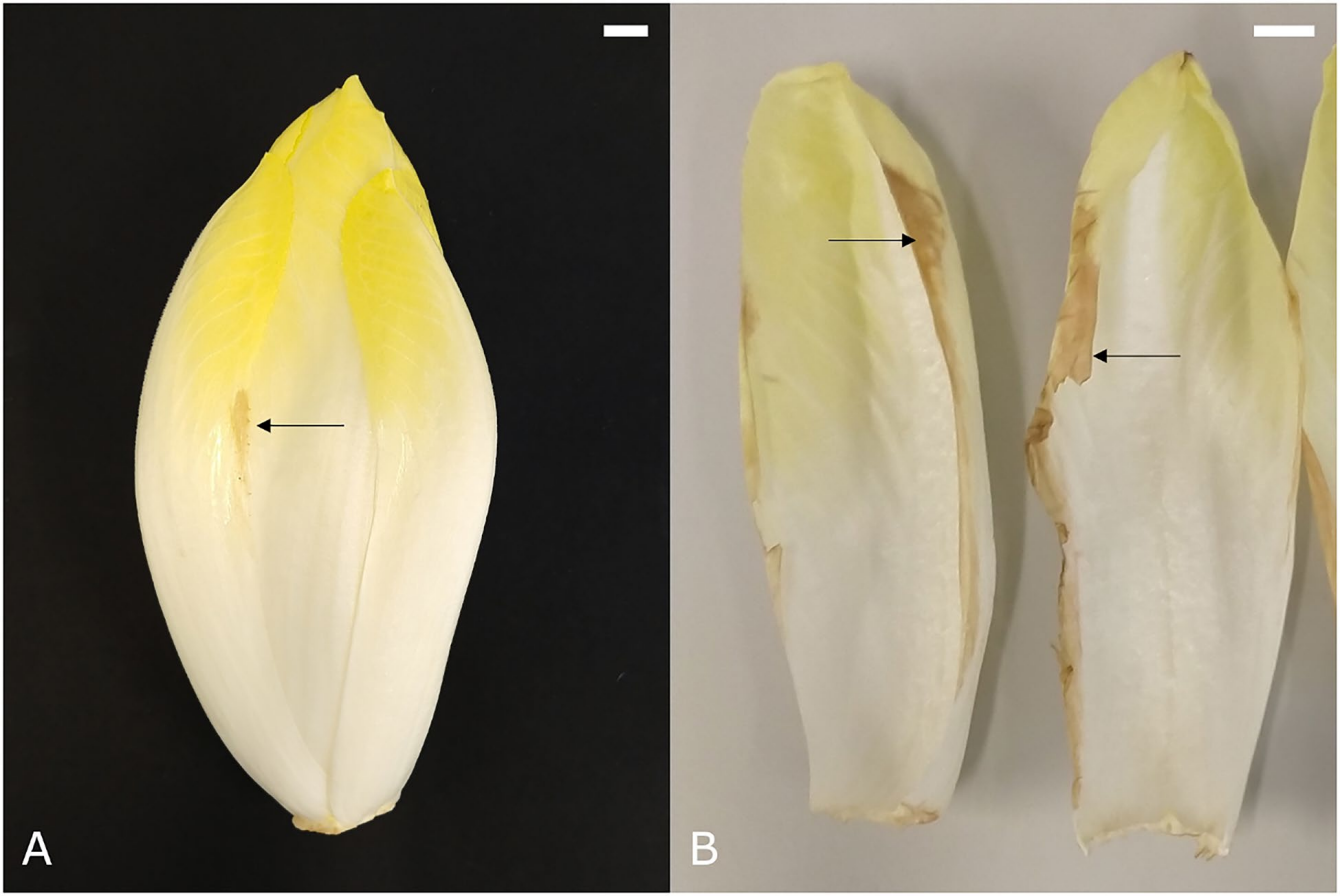
Symptoms of **(A)** mild and **(B)** severe leaf edge browning of the external leaves. Scale bars represent 1 cm.

Chicons with brown edges on the external leaves are encountered rather frequently and symptoms can range from a few brown spots near the edge in the middle of the leaf (Figure 3A) to completely brown and necrotic leaf edges (Figure 3B). This color disorder is usually seen on cultivars with thin outer leaves and develops predominantly postharvest (Reerink, 1994). Leaf edge browning in chicory resembles a number of browning disorders in other crops, such as tipburn in lettuce, which are typically related to cellular disintegration as a result of calcium deficiency in young leaves that have little transpiration (Reerink, 1994; Barta and Tibbitts, 2000). However, the link between leaf edge browning and calcium has not been studied in chicory and in contrast to other crops, it occurs mainly in the oldest leaves of the chicon, which are most exposed to the environment. Alternatively, leaf edge browning could also be linked to leaf dehydration, causing mesophyll cells in the thin leaf margin to collapse, thus leading to browning of the disintegrated tissue (van Kruistum, 1992).

### Less Frequently Encountered Discolorations

#### Point Noir

**Figure 4:**
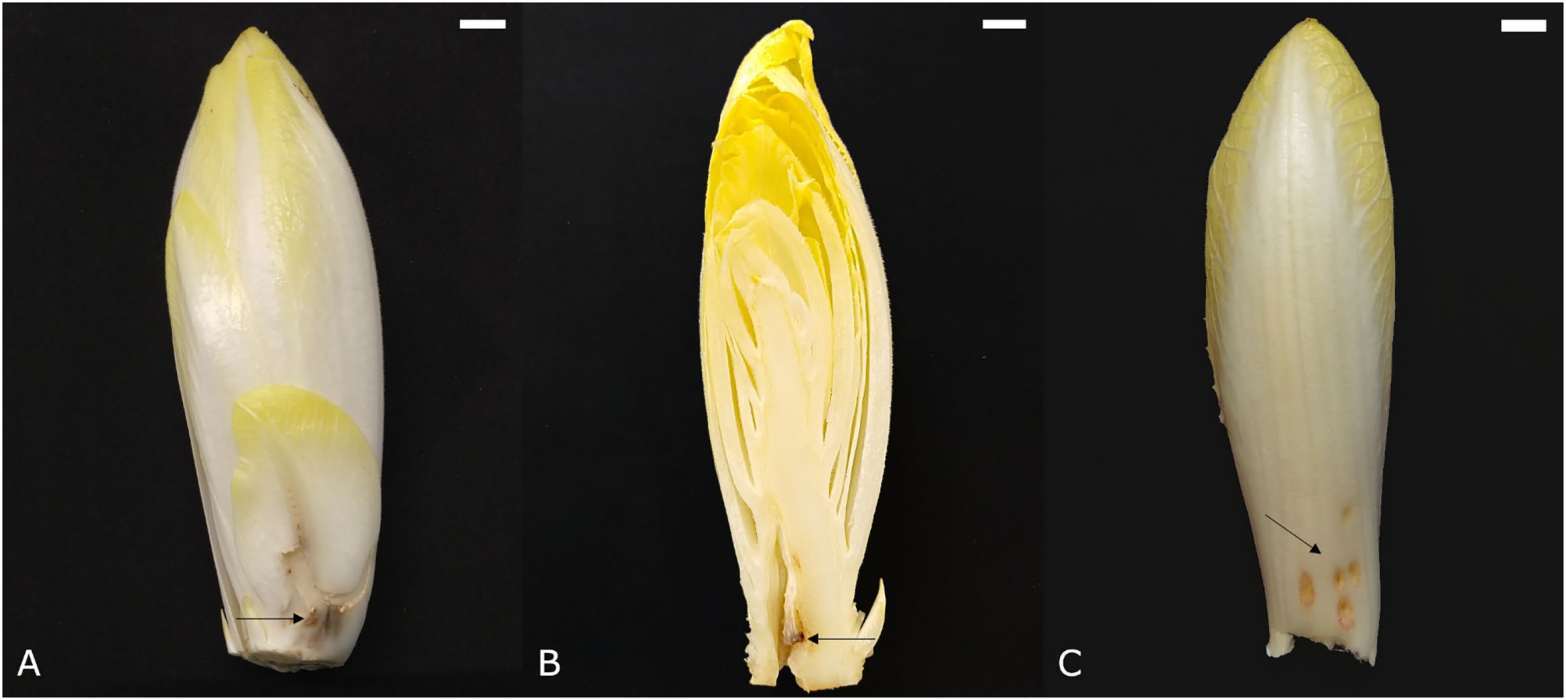
Symptoms of point noir on **(A)** an outer leaf and **(B)** the pith. **(C)** Symptoms of chilling injury. Scale bars represent 1 cm.

*Point noir*, French for “black spot,” refers to the development of a black region of necrotic tissue on the leaves (Figure 4A). Subsequent growth of healthy tissue around the affected spot results in an atypically bent leaf. The *point noir* disorder can also occur in the pith, causing chicon growth to be seriously hampered and resulting in the development of a cavity in the core around the black spot (Figure 4B). *Point noir* typically occurs during early and winter forcing (October–January), becoming more pronounced after prolonged storage of the roots. Its incidence is more outspoken when chicory plants suffered dry conditions during the preceding field cultivation phase. A significant reduction of *point noir* symptoms can be achieved when roots were treated with CaCl_2_ before forcing (van den Broek, 1994).

#### Chilling Injury

Inadequate cooling after chicon harvest can lead to the development of superficial red-brown depressed spots on the abaxial side of leaves (Figure 4C). This disorder is also referred to as low temperature injury and manifests itself predominantly when the harvested chicons are stored at an intermediate temperature (±18°C) for 1–2 days before actual long term storage at 1°C. The symptoms become more apparent when chicons are again exposed to higher temperatures (±15°C). Sensitivity to this disorder is highly variable among cultivars (van Kruistum et al., 1995).

#### Blue or Black Discolored Leaves

**Figure 5:**
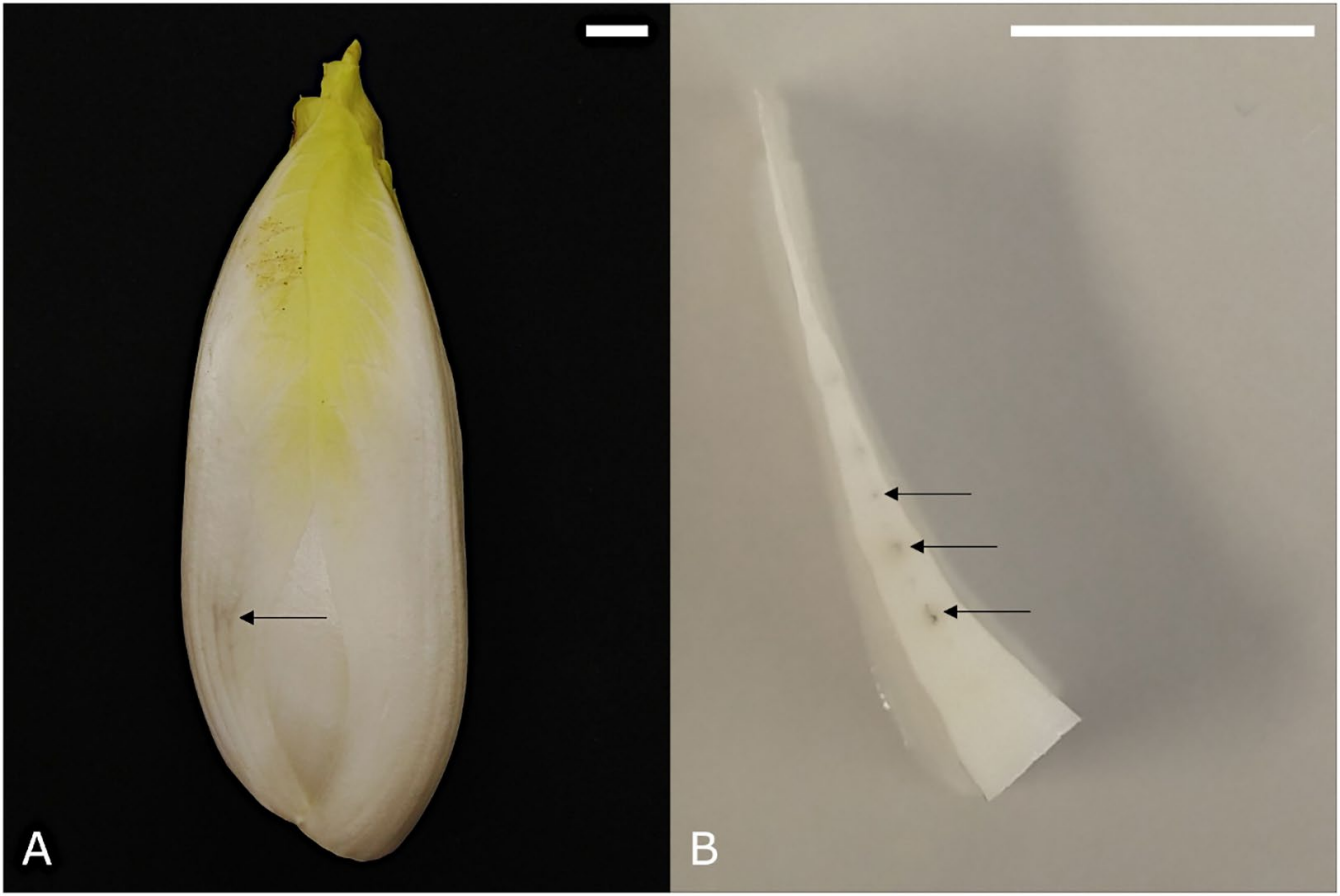
**(A)** Symptoms of black discoloration of a chicon and **(B)** the localization of black pigment in the vascular tissue on a cross section. Scale bars represent 1 cm.

Occasionally, patches of dark discolored tissue can be seen on the leaves of the chicon (Figure 5A). These blue or black discolorations typically occur in the vascular tissue (Figure 5B) and are thought to be caused by excessive uptake of iron due to low soil pH or low calcium content in ferrous, oxygen-deprived soils during root cultivation or soil-covered forcing. The dark pigment likely results from a complexation reaction between iron and polyphenols. This type of discoloration occurs more frequently in traditional soil forced chicory, because the composition of the nutrient solution can be managed more precisely in a hydroponic forcing system (van Kruistum and Zwanepol, 1997).

## External Factors Influencing Color Disorders

The occurrence of chicory discolorations varies considerably throughout the forcing season and between successive years, indicating that root age and environmental conditions during field cultivation and root storage can have a major impact on subsequent chicon quality (Godts et al., 2012). Past research has improved our understanding of the conditions that influence the development of color disorders and has led to the formulation of a range of guidelines for cultivation and postharvest storage practices that can effectively reduce the prevalence of certain disorders. Apart from cultivation conditions and postharvest storage procedures, the genetic differences between cultivars may also account for a lot of the variability in discoloration sensitivity.

### Cultivation Practice and Postharvest Storage

Past research on chicory discolorations has focused primarily on the influence of different cultivation techniques and forcing conditions on the development of color disorders. This has led to the identification of a number of influencing factors and the formulation of cultivation guidelines to minimize discolorations. In general, controlling color disorders requires a balance between yield and product quality, since conditions that promote high yield can jeopardize chicon quality. This is especially true for internal red and leaf edge browning, which are both considerably reduced by harvesting the chicons in time and by using a sufficiently low forcing temperature to prevent rapid chicon growth (Reerink, 1994; Godts et al., 2012). Likewise, the treatment of root heads with CaCl_2_ before forcing has been shown to reduce core browning and hollow pith, but comes at the cost of a slightly reduced yield (van den Broek, 1994; Versluis, 1994). Although climatological conditions and soil properties can seriously influence the sensitivity to color disorders such as tipburn and pinking in lettuce (Olson et al., 1967; Hunter et al., 2017), these factors have not been reported to play an important role in the development of the most prevalent discolorations in chicory. With regard to postharvest storage, it is imperative that chicons are cooled down swiftly after harvest and stored at low temperature to minimize the development of color disorders (Godts et al., 2012). Additionally, storage under modified atmosphere conditions may further reduce the incidence of certain discoloration types, as was shown for internal red and leaf edge browning (Reerink, 1994; Vanstreels et al., 2002).

Table 1 summarizes a number of cultivation, forcing, and postharvest factors that influence sensitivity to internal red, leaf edge browning, and core browning. Some factors are specific for certain discoloration types while others, such as root ripeness, are more general. The influence of forcing and storage practices is more important than the chemical composition of the roots, especially with respect to the development of internal red (Seynnaeve et al., 2000). As a consequence, roots with an identical origin (cultivar/field/harvest date/storage conditions), may develop chicons with varying levels of discoloration when grown in different forcing facilities (van Kruistum, 1992; Coppenolle et al., 2001). Similarly, roots of variable origins will produce chicons with a different sensitivity to discolorations when forced in the same facility (van Kruistum, 1992; Coppenolle et al., 2001).

**Table 1:** Overview of influencing factors during root cultivation, forcing and postharvest storage of witloof chicory on the sensitivity toward the development of internal red, core browning, and leaf edge browning.

	Influencing factor	Discoloration type		
		Internal red	Core browning	Leaf edge browning
Root characteristics	Composition	High N content: ↓ (van Kruistum and Zwanepol, 1997)	High N content: ↑ (van Kruistum, 1992)	
	Diameter	Large: ↑ (van Kruistum, 1992)	Large: ↑ (van Kruistum, 1992; Van Stallen et al., 2005)	Small: ↑ (van Kruistum, 1992)
	Ripeness^*^	Unripe/overripe: ↑ (van Kruistum and Zwanepol, 1997)	Unripe/overripe: ↑ (van Kruistum and Zwanepol, 1997)	Unripe/overripe: ↑ (van Kruistum and Zwanepol, 1997)
	Dry matter content	High DM: ↓ (Reerink, 1994)		
Forcing conditions	Root treatment	Shortening root: ↓ (Coppenolle et al., 2001) Boron treatment: ↓ (Hunter et al., 2017)	CaCl_2_ treatment: ↓ (Versluis, 1994)	
	Temperature^**^	High: ↑ (Coppenolle et al., 2001)		High: ↑ (Reerink, 1994)
	Duration^**^	Long: ↑ (Reerink, 1994)		Long: ↑ (van Kruistum, 1992; van Kruistum and Zwanepol, 1997)
	Nutrient solution	More K^+^ toward end of forcing: ↓ (Reerink, 1994)	More K^+^: ↓ (Reerink, 1993)	More K^+^ toward end of forcing: ↓ (Reerink, 1994)
Postharvest storage	Storage temperature	High: ↑ (Reerink, 1994)		High: ↑ (Reerink, 1994)
	Storage duration	Long: ↑ (Reerink, 1994)		Long: ↑ (Reerink, 1994)
	Atmosphere	10% O_2_ + 10% CO_2_ at 5°C: ↓ (Vanstreels et al., 2002)		10% O_2_ + 10% CO_2_ at 5°C: ↓ (Vanstreels et al., 2002)

↑ = increased sensitivity, ↓ = decreased sensitivity.

* Root ripeness indicates the moment when forcing will result in optimal chicon yield and quality.

** Level of factors should be interpreted relative to standard practice, which may differ with respect to cultivar and time of year.

## Genetic Determinant of Color Disorders

Contrary to the role of cultivation and postharvest factors, the genetic determinant of chicory discolorations has not received much attention to date. It is assumed that the narrow genetic diversity among witloof chicory hybrids may explain the persistence of discoloration problems over many years (de Proft et al., 2003). It has previously been established that discoloration sensitivity varies between cultivars. For example, leaf edge browning tends to occur more often in cultivars with thin and translucent outer leaves. Likewise, chilling injury, internal reddening, and pith browning were shown to be highly cultivar dependent (van Kruistum, 1992; van den Broek, 1994; van Kruistum et al., 1995; Coppenolle et al., 2001; Godts et al., 2012). Although this genetic factor has often been reported, little is known that can explain discoloration differences between cultivars. A QTL analysis showed genetic linkage for pith browning and hollow pith, but not up to the level of single gene resolution (Van Stallen et al., 2005). Future studies that will deploy new genetic tools and use novel genomic resources, could improve our understanding of the genetic regulation of color disorders in chicory (Barcaccia et al., 2016; Bernard et al., 2019; Bogdanović et al., 2019; Aldahak et al., 2021).

## Physiological Causes of Color Disorders

Over the years, a number of physiological causes have been linked with the development of color disorders in chicory, ranging from laticifer rupture to calcium deficiency. Here, we will discuss the most prevalent hypotheses concerning common discoloration types.

### Laticifer Rupture

Laticifer cells are present in approximately 10% of all flowering plants and compose a tubular network filled with milky latex, which contains a broad range of specialized metabolites and defense proteins (Castelblanque et al., 2016). Although its specific function is not entirely clear, latex is thought to play a role in plant defense by creating a physical and chemical barrier against pests. Coagulated latex facilitates wound closure and pest entrapment, while secretory compounds within the latex may have an antibiotic effect (Castelblanque et al., 2020). In chicory, articulated laticifers compose an anastomosing network that is tightly associated with the phloem in the vascular bundles (Vertrees and Mahlberg, 1978). Chicory laticifers contain no phenolic compounds, but exhibit both polyphenol oxidase and peroxidase activity (Mohamed-Yasseen and Splittstoesser, 1990). The absence of phenols may explain the lack of browning in exuded latex after wounding, much in contrast to the immediate browning reaction that occurs in the polyphenol-rich latex of related dandelion species (*Taraxacum* spp.; Wahler et al., 2009). However, the release of latex may elicit a discoloration reaction with the phenolic compounds located in the surrounding mesophyll cells or apoplast. In lettuce, fluctuating water availability in combination with local calcium deficiency leads to overpressure in laticifer vessels, resulting in deformations of the weakened cell walls and leakage of latex into neighboring tissue, which causes the browning phenomenon tipburn (Olson et al., 1967; Tibbitts et al., 1985; Barta and Tibbitts, 2000). Similarly, in growing chicory leaves, rapid water uptake during cell elongation, is thought to cause excessive asymmetrical pressure between laticifers and the surrounding tissue. This can cause laticifer cells to rupture and bleed latex into the intercellular space, causing the elongated red spots that are typical for external red discoloration of the leaves. The rupture of laticifer cells has also been mentioned in the context of internal red and leaf edge browning. However, cells affected by internal reddening are usually situated under the upper epidermis, relatively far away from the laticifer-encased vasculature in the center or toward the abaxial side of the leaf. Moreover, it was found that the latex-exuding functionality of laticifer cells was not compromised in leaves affected by internal red discolorations (Veen, 1999). As for leaf edge browning, the association with laticifer rupture is uncertain, because this disorder occurs typically in the oldest outer leaves, in which the laticifer system has long been developed. Additionally, laticifer cells in chicory are predominantly localized on the abaxial side of the leaf, while brown edge symptoms can occur at both sides (Reerink, 1994).

### Calcium Deficiency

Calcium ions play a critical role in the maintenance of cellular integrity by promoting cell wall rigidity and stability of the cell membranes and the middle lamellae (de Barsy and Bronchart, 1991; White and Broadley, 2003). Long-distance calcium transport to the shoot occurs *via* the xylem and is thereby dependent on transpiration. Subsequently, Ca^2+^ ions are further mobilized predominantly *via* the apoplast and enter the cell passively through Ca^2+^ channels (White and Broadley, 2003). In a range of different crops, an inadequate supply of Ca^2+^ is associated with physiological color disorders in developing tissues that have a low transpiration rate, such as young leaves, enclosed tissues, and storage organs (Den Outer, 1989; White and Broadley, 2003; Adams and Brown, 2007). For example, internal necrosis in potato, which is caused by a calcium deficiency, shows a striking similarity to pith disorders in chicory (Thornton et al., 2020). Furthermore, many leafy vegetables can develop calcium-related discolorations that resemble the symptoms of leaf edge browning in chicons, such as tipburn in lettuce (Reerink, 1994). Chicory roots, which are naturally low in calcium (±2.3 mg g^−1^ DW), are forced under conditions of low root pressure and limited transpiration (Den Outer, 1989; Reerink, 1993). During the first half of the forcing period, translocation of calcium from the nutrient solution to the apical bud is very low and as a result, the growing chicon relies entirely on the limited redistribution of calcium from the taproot (Limami and Lamaze, 1991; Reerink, 1993). Toward the end of the forcing period, when secondary roots have developed and more water can be taken up for rapid cell elongation, calcium translocation from the nutrient solution to the chicon increases, reaching approximately 3.5–5 mg g^−1^ DW in the harvested chicon. This is considerably lower than calcium levels in leaves of other crops, which usually range from 7 to 65 mg g^−1^ DW (Reerink, 1994). Local calcium deficiencies have been proposed to be involved in multiple color disorders in chicory, but are particularly associated with the development of a brown pith (Den Outer, 1989; de Barsy and Bronchart, 1991). A pre-treatment of chicory roots with CaCl_2_ before forcing alleviates the symptoms of brown pith, hollow pith, and point noir, but not of internal red (van den Broek, 1994; Versluis, 1994). Moreover, symptoms of pith browning are initiated during the early forcing stage characterized by low calcium uptake and mobilization, in contrast to the symptoms of internal red and leaf edge, which mainly develop during postharvest storage (Fouldrin and Limami, 1993). Calcium distribution within a chicon is comparable to other crops, with higher Ca^2+^ concentrations in older leaves and lowest Ca^2+^ levels in younger leaves (Veen, 1999). The occurrence of reddening in the medial leaves and leaf edge browning in the old leaves does not correlate with their relatively high calcium content, which makes these disorders less likely to be caused by a calcium deficiency. A dedicated analysis of the calcium content in the leaf edge, upper rib, and lower rib (where reddening usually occurs), revealed a slightly lower calcium content in the lower rib, but no relation with reddening could be deduced (Veen, 1999). However, the incidence of a temporary calcium deficiency in these tissues cannot be ruled out.

### Water Distribution

Both a surplus and a loss of water can play an important role in the development of color disorders in chicory. Excessive uptake of water for cell elongation during the final stage of forcing may lead to pressure imbalances between the continuous laticifer system and the surrounding mesophyll tissue (Reerink, 1994). Consequent laticifer rupture can then facilitate the oxidation of phenolic substrates by polyphenol oxidase, resulting in the elongated red spots that typify external red discolorations. In contrast, both internal red and leaf edge browning are more likely to result from loss of water in the affected tissue, independent of laticifers. Coppenolle et al. (2001) showed that leaves displaying the most intense reddening symptoms also have the highest water potential. After harvest, this makes them more prone to internal water redistribution toward the pith, which has a more negative water potential and keeps growing after harvest (Coppenolle et al., 2001). This process was validated by imposing an external potential pressure on harvested chicory heads by storing them with their base in either distilled water or in a sorbitol solution with a more negative water potential than the pith. This resulted, respectively, in a decrease and increase of the reddening symptoms, indicating that a strong negative water potential of the pith causes strong gradients in water content that can give rise to internal red. Similarly, postharvest storage of chicons detached from or attached to their taproot, resulted in more and less red discoloration of the leaves (Veen, 1999), confirming that water loss from the leaves leads to internal reddening. Finally, loss of water from the outer leaves due to desiccation is likely to be the driving force behind the development of leaf edge browning after harvest (van Kruistum, 1992). Differences in the transpiration rate between cultivars could help to explain the observed variation in sensitivity to leaf edge browning.

### Tissue Tension

The closed conformation of a chicory head imposes some mechanical stress on the inner surface of the medial leaves. According to Veen (1999), tissue tension is maximal just below the upper epidermis and may attribute to the development of internal red discolorations by promoting the development of tears in the tissue just below the adaxial epidermis. Additionally, Gillis et al. (2001) showed that the application of a mechanical load on individual leaves induced red discoloration symptoms corresponding to the pattern of stress distribution within the leaves. Presumably, tissue tension can lead to loss of cellular integrity, resulting in unwanted discolorations in the affected tissue.

## Biochemistry of Discoloration in Chicory and Related Species

Many browning reactions in fruits and vegetables are attributed to the enzymatic activity of polyphenol oxidases (PPOs; Martinez and Whitaker, 1995; Tomás-Barberán and Espín, 2001; Yoruk and Marshall, 2003). PPOs and their phenolic substrates, which are localized in the plastids and the vacuole, respectively, can be decompartmentalized when cellular integrity is lost, e.g., due to senescence, dehydration, or mechanical trauma. This enables subsequent enzymatic oxidation and discoloration of the affected tissue (Yoruk and Marshall, 2003). Although this mechanism has long been thought to cause discolorations in witloof chicory, little research has been done to validate this hypothesis. The discoloration process in lettuce, however, has received far more attention in recent years and could serve as a paradigm for chicon discolorations (Hunter et al., 2017; Saltveit, 2018). It is worth noting that not all browning reactions of fresh produce are associated with PPO activity. For example in lettuce, researchers showed that the formation of yellow-brown lettucenin sesquiterpenes contributed significantly to the browning of cut tissues (Mai and Glomb, 2014). Nevertheless, phenolic oxidation by PPO remains a major source of discolorations in fruit and vegetable products and is therefore an interesting target for research on color disorders in chicory.

### PPO in Plants

Polyphenol oxidase proteins are type-III copper enzymes that are nearly ubiquitous in higher plants. They are typically localized in the plastids and can be either soluble or membrane-bound (Martinez and Whitaker, 1995; Kampatsikas et al., 2019). Much of the research on PPOs in plants has focused on their negative impact on fresh produce quality e.g., browning reactions in apple, potato, and lettuce (Yoruk and Marshall, 2003). However, PPOs play an important role in plant defense by contributing to wound healing and immune responses against pathogens and pests (Kampatsikas et al., 2019). Additionally, some PPO isoforms have a specific function in the biosynthesis of specialized metabolites. The distribution of the *PPO* gene family is highly variable among plant taxa. Different isoforms show diverse substrate specificities and their expression is subject to both temporal and spatial regulation in response to environmental conditions (Tran et al., 2012; Dirks-Hofmeister et al., 2014). This flexibility allows PPO enzymes to fulfill a range of physiological functions in different tissues during different developmental stages.

### PPO Reaction Mechanism and Phenolic Substrates in Chicory

Phenolic compounds are synthesized by the phenylpropanoid pathway, of which phenylalanine lyase (PAL) is the first and rate-determining enzyme (Hunter et al., 2017). Generally, the amount of *PAL* transcript and PAL activity is low, but expression can be induced by wounding or exposure to hormonal levels of ethylene (Tomás-Barberán and Espín, 2001; Kang and Saltveit, 2003). In chicory, phenolic compounds are highly abundant in parenchyma cells and to a lesser extent in the vicinity of the vascular tissue (Reerink, 1994). Interestingly, chicory laticifers contain no phenolic compounds as evidenced by the lack of browning in exuded latex (Mohamed-Yasseen and Splittstoesser, 1990). PPO catalyzes the first step in the oxidation of phenolic compounds to complex brown polymers or melanins, which are typically associated with color disorders in fresh produce. Depending on the type of PPO, the enzyme can catalyze the oxidation of monophenols and/or *o*-diphenols to *o*-quinones in the presence of molecular oxygen (Kampatsikas et al., 2019). These highly reactive and colored *o*-quinones can then lead to the formation of melanin *via* non-enzymatic reactions with different cellular components, such as proteins, amino acids, and other quinones (Martinez and Whitaker, 1995; Yoruk and Marshall, 2003).

Substrate specificity of PPOs toward different phenolic compounds varies considerably between species (Yoruk and Marshall, 2003). For lettuce PPO, the highest substrate specificity was observed for caffeic acid and chlorogenic acid, resulting in the formation of a pink and green quinone, respectively (Saltveit, 2018). In etiolated endive, the relative contribution of caffeic acid derivatives to the total phenolic content was shown to be increased in comparison to that of regular endive, which also developed less enzymatic discolorations than its dark-grown counterpart (Reerink, 1994). Caffeic acid and its derivatives also make up a major part of the total phenolic content in chicory, thus identifying them as potential precursors in the development of color disorders in chicons (Hanotel et al., 1995). The red color of several discoloration types of chicory may be the result of an interaction between PPO reaction products and free amino acids such as proline and tryptophan. Accordingly, the amount of free amino acids in chicory heads was found to be strongly correlated to the incidence of internal red during postharvest storage (Godts et al., 2012). Alternatively, the red pigment could result from the interaction between caffeic acid quinone and cellulose in the cell walls, which results in a stable pink complex as described in lettuce (Saltveit, 2018).

### Strategies to Reduce Enzymatic Discoloration

Several approaches targeting the PPO enzyme family have proven to be successful in reducing browning reactions in fruits and vegetables. Since the rate of browning depends on both PAL and PPO activity, as well as substrate availability, O_2_ concentration, pH, and temperature, each of these factors can be managed in order to reduce the occurrence of color disorders (Martinez and Whitaker, 1995). As such, several postharvest approaches have proven to be successful in diminishing oxidative discoloration in fresh produce. This includes physical methods such as heat treatments, modified atmosphere packaging, storage at low temperature and irradiation, and chemical treatments such as the application of reducing agents, copper-chelating agents, natural anti-browning compounds, and acidification (Moon et al., 2020). In chicory, successful postharvest interventions have mainly aimed to tackle color disorders *via* the use of a sufficiently low storage temperature and modified atmosphere packaging (see also Table 1). For example, the combination of 10% CO_2_ and 10% O_2_ at 5°C led to a maximal reduction of internal red discoloration and additionally reduced symptoms of leaf edge browning, rot and continuation of pith growth (Vanstreels et al., 2002). One study also indicated the feasibility of reducing red discoloration of the chicon base using a heat-shock treatment of 2 min at 42°C after harvest (Jung and Kang, 2014). This reduction is thought to be achieved by the suppression of wounding-induced PAL synthesis due to a preferential synthesis of heat-shock proteins, thereby reducing accumulation of phenolic compounds and subsequent base discoloration (Kang and Saltveit, 2003). In practice, the only strategy that is consistently used to decrease discoloration incidence in chicons, is postharvest storage at low temperature, which is also essential for good shelf-life. Storage under modified atmosphere, however, is not standard practice and requires additional investments in specialized equipment.

Other than postharvest interventions, breeding strategies addressing the phenylpropanoid or PPO biosynthesis pathway could also be an effective solution (Hunter et al., 2017). There have been several successful attempts to silence *PPO* expression and thereby reduce discolorations in fresh produce, resulting in commercial applications such as Arctic® apple and Innate® Potato (Richael, 2021; Stowe and Dhingra, 2021). However, it is possible that the suppression of *PPOs* results in an increased susceptibility toward disease, due to their role in plant defense (Kampatsikas et al., 2019). Additionally, the PPO enzyme is not necessarily responsible for the full array of discolorations in a particular crop. Both in lettuce and chicory, a direct link between PPO activity, phenolic content, and the degree of discolorations could not always be observed (Jung and Kang, 2014; Saltveit, 2018). This may limit the applicability of *PPO* silencing in species were these enzymes play an important role in plant defense or when other specialized metabolites are at play. Alternatively, breeding efforts could target PAL or an enzyme downstream of PAL in the biosynthesis pathway of PPO substrates (Hunter et al., 2017). An improved understanding of the discoloration biochemistry and the nature of the different discoloration compounds in witloof chicory would be invaluable to identify novel targets for future breeding efforts to prevent color disorders.

## Author Contributions

ID performed literature research and took pictures. ID and BP wrote the manuscript. YC, TD, and AG provided feedback and corrected the manuscript. All authors approved the final manuscript.

## Funding

This work was financially supported by Flanders Innovation & Entrepreneurship (VLAIO) through the LA-traject grant “ChiQon” (HBC.2018.2218).

## Conflict of Interest

The authors declare that the research was conducted in the absence of any commercial or financial relationships that could be construed as a potential conflict of interest.

## Publisher’s Note

All claims expressed in this article are solely those of the authors and do not necessarily represent those of their affiliated organizations, or those of the publisher, the editors and the reviewers. Any product that may be evaluated in this article, or claim that may be made by its manufacturer, is not guaranteed or endorsed by the publisher.
